# Not just who, but how many: the importance of partner abundance in reef coral symbioses

**DOI:** 10.3389/fmicb.2014.00400

**Published:** 2014-08-04

**Authors:** Ross Cunning, Andrew C. Baker

**Affiliations:** Department of Marine Biology and Ecology, Rosenstiel School of Marine and Atmospheric Science, University of MiamiMiami, FL, USA

**Keywords:** coral, *Symbiodinium*, symbiont density, cell ratio, normalization, symbiosis regulation, benefits and costs, density dependence

## Abstract

The performance and function of reef corals depends on the genetic identity of their symbiotic algal partners, with some symbionts providing greater benefits (e.g., photosynthate, thermotolerance) than others. However, these interaction outcomes may also depend on partner abundance, with differences in the total number of symbionts changing the net benefit to the coral host, depending on the particular environmental conditions. We suggest that symbiont abundance is a fundamental aspect of the dynamic interface between reef corals and the abiotic environment that ultimately determines the benefits, costs, and functional responses of these symbioses. This density-dependent framework suggests that corals may regulate the size of their symbiont pool to match microhabitat-specific optima, which may contribute to the high spatiotemporal variability in symbiont abundance observed within and among colonies and reefs. Differences in symbiont standing stock may subsequently explain variation in energetics, growth, reproduction, and stress susceptibility, and may mediate the impacts of environmental change on these outcomes. However, the importance of symbiont abundance has received relatively little recognition, possibly because commonly-used metrics based on surface area (e.g., symbiont cells cm^-2^) may be only weakly linked to biological phenomena and are difficult to compare across studies. We suggest that normalizing symbionts to biological host parameters, such as units of protein or numbers of host cells, will more clearly elucidate the functional role of symbiont abundance in reef coral symbioses. In this article, we generate testable hypotheses regarding the importance of symbiont abundance by first discussing different metrics and their potential links to symbiosis performance and breakdown, and then describing how natural variability and dynamics of symbiont communities may help explain ecological patterns on coral reefs and predict responses to environmental change.

## INTRODUCTION

Reef corals engage in symbiosis with single-celled dinoflagellate algae in the genus *Symbiodinium*, from which they acquire photosynthetic products that support most or all of their energetic needs (Muscatine and Porter, 1977) and help them build calcium carbonate skeletons that form the foundation of coral reefs (Allemand et al., 2011). The future growth and persistence of these ecosystems therefore depends on the integrity of coral-algal symbiosis under anthropogenic climate change. Coral bleaching—the breakdown of symbiosis that can lead to coral mortality—is predicted to occur with greater frequency and intensity due to rising sea surface temperatures (Hoegh-Guldberg et al., 2007; Baker et al., 2008), although individual responses may vary greatly in space and time. Investigating the basic functional biology of coral-algal symbiosis has helped us understand this variability and improves our ability to forecast the potential fates of coral reefs under climate change.

The functional response of the coral “holobiont” (the animal host and its symbionts) is known to depend on the genetic composition of its symbiotic algal community. Different taxa within the genus *Symbiodinium* (Pochon and Gates, 2010) vary in their physiological properties, and certain taxa, particularly members of clade D, are heat-tolerant (Rowan, 2004), conferring increased resistance to thermal stress on their coral hosts (Rowan et al., 1997; Glynn et al., 2001; Berkelmans and van Oppen, 2006; LaJeunesse et al., 2010; McGinley et al., 2012; Cunning and Baker, 2013). Other types, including members of clade C, may provide corals with more fixed carbon (Cantin et al., 2009), enabling faster growth (Little et al., 2004; Jones and Berkelmans, 2010). Symbiont taxa also differ in their ability to acquire inorganic nutrients (Baker et al., 2013) and combat oxidative stress (McGinty et al., 2012). Together, these differences likely help explain significant variation in growth, performance, and stress susceptibility among corals hosting different symbiont types.

However, the expressed phenotype of coral holobionts likely also depends on the abundance of algal symbionts within coral tissues, and not just their genetic identity. Indeed, symbiont population density may directly influence the costs, benefits, and outcomes of all symbiotic interactions (Holland et al., 2002, 2004). In corals, symbiont abundance is variable in space and time (Fagoonee et al., 1999; Fitt et al., 2000), and may strongly influence most, if not all, aspects of reef coral physiology, including nutrient cycling (Wooldridge, 2009), light absorption (Enríquez et al., 2005), and stress response (Nesa and Hidaka, 2009; Nesa et al., 2012; Cunning and Baker, 2013). However, despite the potential importance of symbiont abundance, its specific role in determining coral functional responses is poorly understood and often overlooked. This may be due in part to the preoccupation of recent work with genetically identifying (rather than quantifying) symbionts. Moreover, the different metrics used to normalize symbiont abundance (e.g., per unit area, mass, volume, protein, or cell) may not all have equal relevance to symbiosis physiology, potentially obscuring important functional relationships (Edmunds and Gates, 2002), and precluding useful comparisons across species and studies.

While most recent studies measure symbiont abundance only to diagnose coral bleaching, earlier studies also focused on understanding how symbiont populations are regulated and controlled (Muscatine and Pool, 1979; Falkowski et al., 1993; Jones and Yellowlees, 1997). Although these studies were primarily concerned with the mechanisms by which a particular abundance is maintained, its subsequent influence on coral physiology and function received less attention. Some studies have evaluated impacts of symbiont abundance on photosynthesis and respiration (Hoegh-Guldberg and Smith, 1989; Hoogenboom et al., 2010), while others have explored its potential physiological impacts using either conceptual (Wooldridge, 2013) or modeling approaches (Anthony et al., 2009; Terán et al., 2010; Cunning, 2013), concluding that symbiont abundance can have fundamental impacts on symbiosis. Here, we advance the view that symbiont abundance is much more than just an indicator of bleaching during stress; it is an integral determinant of holobiont physiology and mediator of symbiosis function that underlies critical variation in symbiosis biology and ecology.

## MEASURING SYMBIONT ABUNDANCE

Many techniques and metrics have been employed to measure the abundance of algal symbionts in cnidarian hosts. In corals, the most commonly used metric is the number of symbiont cells per unit surface area of coral skeleton (cells cm^-2^). Measuring this typically involves extracting intact *Symbiodinium* cells from living corals [e.g., using a Water Pik (Johannes and Wiebe, 1970) or airbrush], counting them with a hemocytometer, and normalizing cell numbers to skeletal surface area. This method is inexpensive but labor-intensive and requires sacrificing several square centimeters or more of coral tissue. The accuracy and precision of this metric depends on complete extraction of symbionts from the skeleton, the breakup of coral mucus to ensure an even distribution of symbionts in the hemocytometer counting field, and accurate measurement of skeletal surface area, all of which can be difficult to achieve without large and compounding errors (Johannes and Wiebe, 1970; Edmunds, 1994; Veal et al., 2010).

Areal symbiont abundance metrics also provide no information about the coral animal inhabiting the same area, which is problematic since coral tissue biomass varies considerably among coral species, colonies, and over time (Fitt et al., 1993; Brown et al., 1999; Fitt et al., 2000; Edmunds and Gates, 2002; Thornhill et al., 2011). Therefore, although different corals may host similar numbers of symbionts per square centimeter of skeleton, these symbionts may be contained within different amounts of host tissue and consequently may function differently. Therefore, normalizing symbiont abundance by area may obscure important functional variation among symbioses related to differences in host tissues (Edmunds and Gates, 2002), emphasizing the need for metrics that better reflect the abundance (or size) of both interacting partners, i.e., a “symbiont to host ratio” (Douglas, 1985). Other metrics address this issue by normalizing symbiont abundance to host-associated biological units instead of areal units.

The number of polyps has been occasionally used to normalize symbiont abundance (Muscatine et al., 1991; Jones and Yellowlees, 1997), although differences in polyp size, structure, and density among coral taxa may prevent useful comparisons of symbiont abundance per polyp (Edmunds and Gates, 2002). Other metrics that are more comparable across taxa include symbiont cells per unit mass (Fitt, 1982), or, more commonly, per unit protein. For protein normalization, researchers either measure total (animal and algal) protein (Saunders and Muller-Parker, 1997; Shick et al., 1999; Edmunds and Gates, 2002; Anthony and Hoegh-Guldberg, 2003; Hoogenboom et al., 2010), or physically separate animal and algal fractions to measure only animal protein (Muller-Parker, 1985; Muller-Parker et al., 1994; Hawkins et al., 2013). Protein is then quantified using the Bradford Assay (Bradford, 1976) and used to calculate symbiont abundance (from cell counts, as above) as cells per mg protein. While this metric provides information about both algal and coral partners, it also has limitations. First, a total protein denominator does not provide a true symbiont to host ratio as it includes algal-derived protein [∼10–13% in anemones (Saunders and Muller-Parker, 1997) and corals (Douglas, 1985)]. Using only animal protein as a denominator theoretically overcomes this issue, although common procedures for mechanically separating algal and animal tissues (i.e., centrifugation) are not fully effective (Douglas and Smith, 1983), leading to considerable error in these metrics. Moreover, these techniques are additionally hampered by issues of incomplete tissue removal from the skeleton, which may be even greater for corals with thicker tissue (Edmunds, 1994) or perforate skeletons.

Symbiont abundance has also been measured by volume (e.g., algal volume as a percent of host cell volume or per mg protein) in green *Hydra* symbioses (Douglas and Smith, 1983, 1984). However, this metric is not amenable to coral symbioses because symbionts occupy nearly 100% of the host cell volume (Muscatine et al., 1998). Moreover, volume estimation relies on assumptions of cell shape and size that are likely incorrect (Douglas, 1985).

To overcome problems associated with volume ratios and ineffective separation of algal and host tissues, the amount of chlorophyll *a* per unit protein (e.g., μg chl a per μg protein) of intact tissues has also been proposed as a useful symbiont to host ratio for diverse invertebrate-algal symbioses (Douglas, 1985). A similar metric of chlorophyll *a* normalized to tissue ash-free dry weight (AFDW) has been used for corals (Grottoli et al., 2004, 2006). However, because symbionts comprise 5–12% of coral AFDW (Porter et al., 1989) and chlorophyll *a* content varies widely per symbiont cell (Chang et al., 1983), this metric may not reflect symbiont abundance so much as the photosynthetic capacity of the symbiosis. As such, it may still provide useful information, and has the advantages of being rapidly and reliably calculated, requiring only small amounts of tissue, and being comparable across diverse symbiotic associations (Douglas, 1985).

Symbiont abundance has also been normalized to host cell numbers. In *Hydra*, the mean number of symbiont cells within a single host digestive cell is a commonly used metric of density (Douglas and Smith, 1984). A similar cell-specific density (CSD) in corals indicates the average number of symbionts within a symbiont-containing gastrodermal cell, which typically has a value between 1 and 2 (Muscatine et al., 1998). However, because corals also contain many non-symbiotic cell types that are not counted in the CSD, this metric is clearly decoupled from tissue- and colony-level phenotypes. Indeed, an increase in CSD can occur simultaneously with major declines in overall symbiont abundance, measured as cells per mg protein (Shick et al., 1999).

More recently, the abundance of symbionts relative to the total number of host cells at the tissue or colony level has been measured using quantitative PCR (qPCR; Mieog et al., 2009; Cunning and Baker, 2013). This technique involves amplification of specific target gene loci in both the symbiont and the host to calculate a ratio of the total number of symbiont cells to host cells (S/H cell ratio). Bulk genomic DNA can be extracted from an intact coral fragment, which overcomes the problems of incomplete tissue removal and fractionation that introduce inaccuracy in other metrics. Moreover, very small tissue samples (0.25 cm^2^ or less) can be used for this analysis, enabling repeated sampling of living coral fragments over time. Most importantly, because this technique enumerates symbionts genetically instead of visually, it can distinguish among different symbiont types in mixed communities at any level of taxonomic resolution. This is of fundamental importance, because the overall function of a symbiont community depends quantitatively on its composition (Loram et al., 2007; Cunning, 2013), and many corals may harbor multiple symbiont types (Silverstein et al., 2012).

Because cells are the fundamental unit of biological organization, standardizing the abundance of symbiont cells to host cells using qPCR may represent the best current approximation of a “symbiont to host ratio” (*sensu* Douglas, 1985). However, as with other techniques, there are drawbacks. These include higher variability among technical replicates than is associated with areal measurements (Mieog et al., 2009) due to the logarithmic error inherent in qPCR. In addition, calculation of absolute S/H cell ratios from qPCR data requires normalizing fluorescence intensity (if different reporter dyes are used) and estimating DNA extraction efficiency and gene copy numbers for target loci (Mieog et al., 2009; Cunning and Baker, 2013; Angly et al., 2014). Primer and probe sequences must also be carefully designed to match target sequences and mismatch non-target sequences (Cunning and Baker, 2013), and some prior knowledge of the symbiont diversity present in a sample is required to select appropriate assays. However, once assays have been developed and validated, they enable higher-throughput data collection relative to methods based on cell counts and surface area, as well as quantitative characterization of the genetic composition of the symbiont community. To date, qPCR assays have been developed to quantify *Symbiodinium* in clades B, C, and D in several coral host species (Mieog et al., 2009; Cunning, 2013; Cunning and Baker, 2013; Silverstein et al., 2014), which can be easily adapted for use in any laboratory with a qPCR platform.

Other genetic techniques for quantifying mixed symbiont assemblages include “FISH-Flow,” which utilizes fluorescence *in situ* hybridization and flow cytometry in tandem to count different symbiont types (McIlroy et al., 2014), and next-generation sequencing (NGS; Kenkel et al., 2013). While their application to coral symbiont communities has only just begun, NGS approaches have the power to recover a more complete picture of community diversity, including the rare biosphere (Quigley et al., 2014), and require no prior taxonomic knowledge. However, while these approaches can estimate relative proportions of different symbiont types, these data are subject to numerous quantitative biases (Amend et al., 2010) and must still be normalized to surface area or other host parameters to quantify symbiont abundance. However, further development of quantitative NGS approaches using appropriate markers for both coral and *Symbiodinium* partners may enable calculation of symbiont to host ratio metrics that identify and quantify all members of the community in a biologically relevant way.

## IMPLICATIONS OF DIFFERENT METRICS OF SYMBIONT ABUNDANCE

Depending on which metric is used to quantify symbiont abundance, different aspects of symbiosis structure and function may be revealed (or obscured). For example, Muller-Parker et al. (1994) found that nutrient enrichment increased the number of symbiont cells per cm^2^ while cells per mg protein remained constant. In contrast, in response to low light, Anthony and Hoegh-Guldberg (2003) found no change in symbiont cells per cm^2^ but more than double the number of cells per mg protein. Similarly, Edmunds and Gates (2002) found that different coral colonies had the same number of symbionts per cm^2^, but significantly different abundances normalized to protein.

**FIGURE 1:**
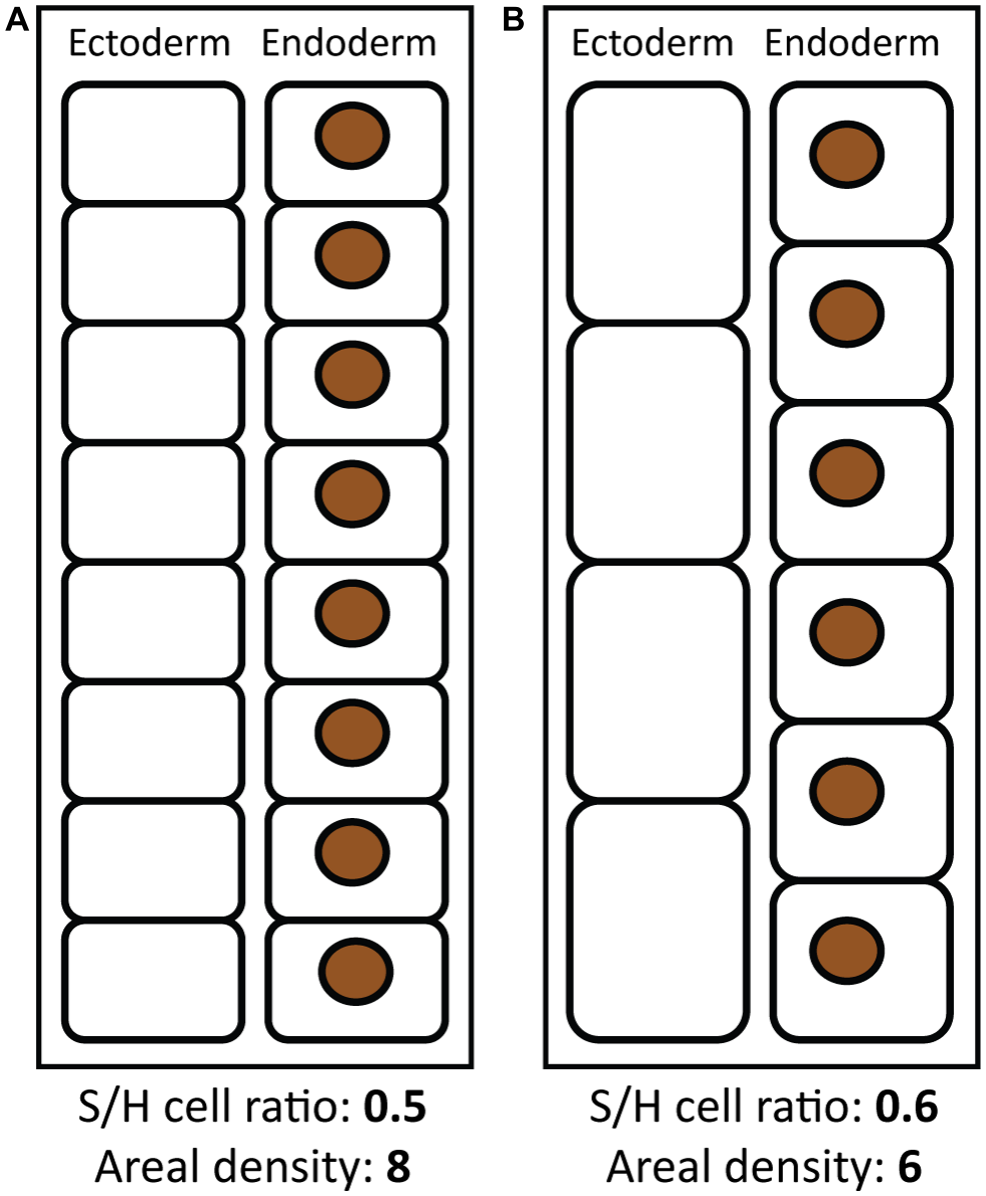
**Coral tissue architecture and different metrics of symbiont abundance.** Two scenarios of coral and symbiont tissue architecture are shown, theoretically representing coral tissues in winter **(A)** and summer **(B)**, with corresponding values of the symbiont to host cell ratio and areal symbiont density. Black rounded rectangles represent coral host cells (comprising ectoderm and endoderm tissue layers) and brown circles represent symbiont cells. Areal symbiont density is higher in **(A)**, while the symbiont to host cell ratio is higher in **(B)**, showing how these metrics may change in opposite ways depending on tissue architecture. Schematics are not to scale and are meant to illustrate conceptual differences between different metrics.

Differences among these metrics are likely the result of a dynamic vs. fixed quantity in the denominator. When symbionts are normalized to a dynamic unit (host protein, cells, etc.), their abundance is also influenced by changes in these units. Therefore, changes in coral tissue architecture may produce different patterns in different metrics of symbiont abundance (**Figure 1**). For example, as environmental conditions change from winter into summer, coral tissues become thinner (Barnes and Lough, 1992; Brown et al., 1999; Fitt et al., 2000; Thornhill et al., 2011), which may involve a loss of both symbiont and host cells on an areal basis. Decreased heterotrophy in summer (Ferrier-Pagès et al., 2011) may also reduce numbers of host prey-capture cells such as cnidocytes and mucocytes, but increased reproduction in summer may increase the number of host gametocytes and mesenterial cells. Higher summer temperatures may also increase respiration and host cell catabolism. Changes in cellular architecture as a result of these processes (e.g., **Figures 1A,B**) might lead to a greater net loss of host cells relative to symbionts, resulting in a reduction in symbionts per cm^2^, but an increase in the S/H cell ratio (**Figure 1**). Indeed, areal symbiont density tends to decrease in the summer (Stimson, 1997; Brown et al., 1999; Fagoonee et al., 1999; Fitt et al., 2000), while the S/H cell ratio may increase (Cunning and Baker, 2013).

Since these metrics provide different information, it is important for researchers to select the most relevant metric. For research focused primarily on interactions with the physical environment (e.g., the interception of light by symbionts), it may be appropriate to normalize symbiont abundance to a physical unit of area. Because light is measured on an areal basis (e.g., μmol quanta m^-2^ s^-1^, or W m^-2^ s^-1^), an areal metric of symbiont abundance may be most appropriate for understanding relationships between symbionts and light. Alternatively, because coral tissues and light fields are three-dimensional, the abundance of symbionts per unit volume may be even more informative (Terán et al., 2010).

In contrast, for research focused primarily on biological interactions between symbionts and hosts, it may be more useful to normalize symbiont abundance to a host-related biological unit (i.e., a “symbiont to host ratio”; Douglas, 1985). The currencies of host-symbiont interactions are metabolites and cellular signaling molecules, which are produced and received by cells as fundamental biological units. Therefore, measuring the abundance of symbionts relative to host cells (or other biological units, e.g., biomass, protein) may be more informative and relevant for research concerned with these interactions. For example, in one study of bleaching and recovery, symbiont abundance per unit area had recovered to pre-bleaching levels within months, but tissue biomass, proteins, and lipids per unit area remained lower than pre-bleaching levels (Fitt et al., 1993). In this case, recovered corals might be expected to function differently from their pre-bleaching state, although areal symbiont abundance metrics would not reveal any difference. Meanwhile, symbiont abundance normalized to a biological parameter might reveal important differences indicative of functional variation.

These issues demonstrate the importance of normalizing data in a way that is relevant to the research question and the response variable of interest. In phototrophic symbioses such as corals, the physical interactions between symbionts and light and the biological interactions between symbionts and hosts are fundamentally linked. Therefore, measuring the number of symbionts normalized to both physical and biological units would provide the most comprehensive information regarding symbiosis function. However, if only one type of metric is to be used, normalizing symbiont abundance to dynamic biological units, rather than static physical units, may be more generally relevant to the physiology and function of coral-algal symbioses (Edmunds and Gates, 2002).

## EFFECT OF SYMBIONT ABUNDANCE ON SYMBIOSIS FUNCTION

Symbiont abundance is an important factor shaping coral tissue microhabitat, resource availability, and symbiont physiology, which in turn determine the overall costs and benefits of symbiosis (Holland et al., 2002, 2004). In corals, both photosynthesis and photo-oxidative stress depend on the light fields that individual *Symbiodinium* experience (Powles, 1984), which are directly modified by the surrounding symbionts (Enríquez et al., 2005; Terán et al., 2010). When symbiont abundance is low, each cell receives more light; as their abundance increases, self-shading reduces light such that symbionts may only receive 10% of the incident light at the colony surface (Kaniewska et al., 2011; Wangpraseurt et al., 2012). Because light absorption takes places within a three-dimensional coral tissue matrix, the magnitude of self-shading is likely a function of symbiont abundance per unit volume, and has been implemented this way (as cells per mm^3^) in modeling these dynamics (Terán et al., 2010).

**FIGURE 2:**
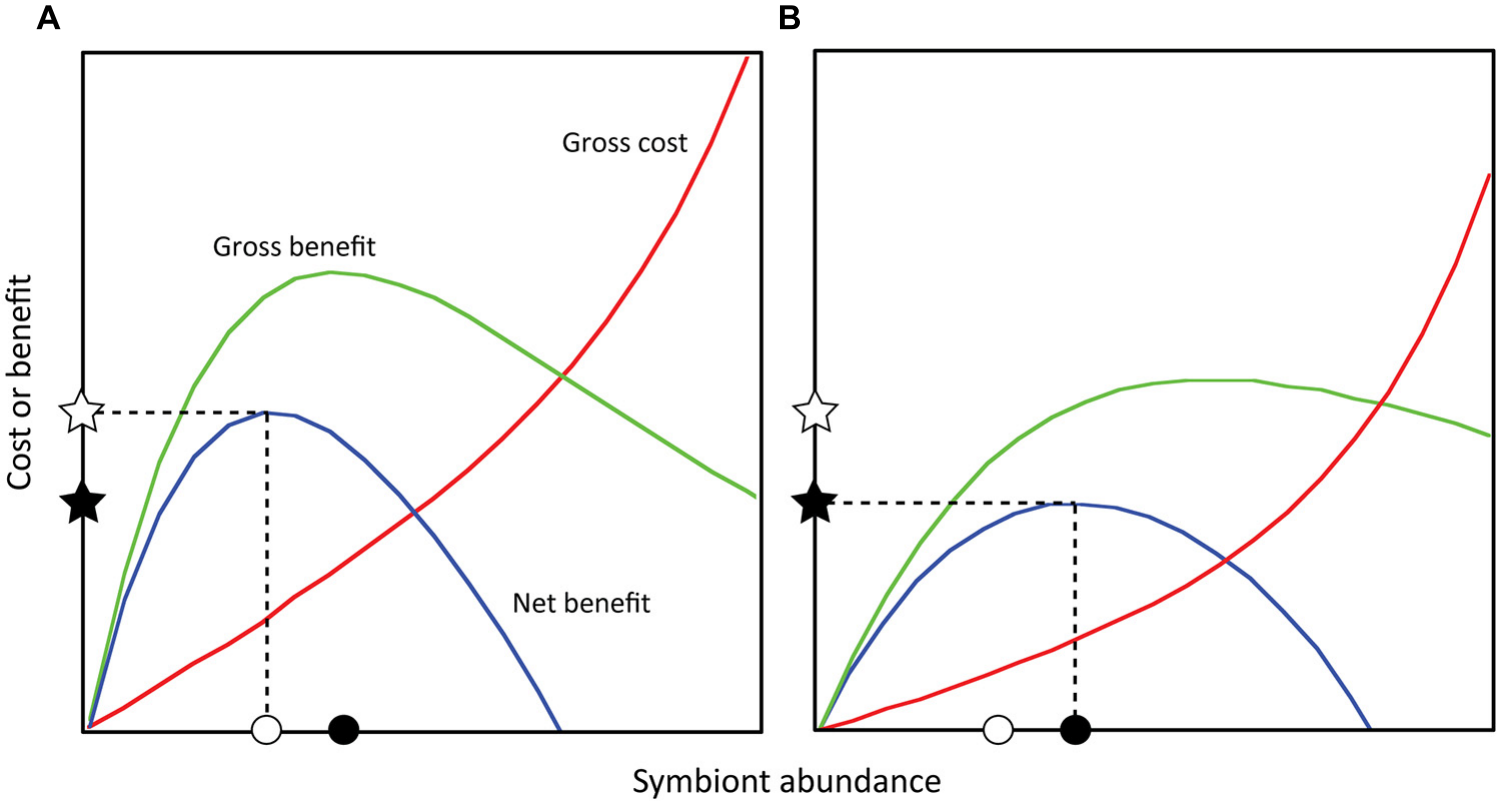
**Theoretical costs and benefits to the coral host as a function of symbiont abundance.** Net benefit equals the gross benefit minus gross cost, and the point at which net benefit is maximized is defined as the optimal symbiont abundance for the coral (Cunning, 2013; *sensu* Holland et al., 2002). Different sets of abiotic (light and temperature) or biotic factors (coral and symbiont type) will alter these functions (e.g., **A** vs. **B**) such that a particular optimal abundance exists for a particular set of conditions. Note that in **(A)**, the optimal symbiont abundance is lower than in **(B)**, but the corresponding net benefit is higher. Within either set of conditions, symbiont abundances above or below the optimum result in decreasing net benefit. Net benefits may be predictive of energetic status, growth rates, or reproductive output.

While incident light may be directly influenced by symbiont abundance, light absorption and quenching involve additional layers of photobiology, and downstream impacts on symbiosis function are further mediated by host-symbiont cellular interactions. Nevertheless, these complex outcomes may still be linked to symbiont abundance and illustrated within a conceptual framework (**Figure 2**). For example, if each symbiont provides some photosynthate, increasing symbiont abundance will increase the total photosynthate received (i.e., the gross benefit to the coral). However, at high abundances, self-shading and/or carbon-limitation may reduce photosynthesis in each cell, causing gross benefit to decline (**Figure 2**). This relationship is supported empirically by P:R ratios in corals that initially increase as a function of symbiont abundance (per mg protein) and subsequently decline (Hoogenboom et al., 2010). Importantly, the impact of photosynthate delivery on the coral depends on the amount of coral tissue receiving it, suggesting that symbiont abundance may better predict gross benefit when normalized to host biological parameters (e.g., protein, cell).

Another outcome linked to symbiont abundance is the energetic cost to the host of maintaining symbionts (Douglas and Smith, 1983). These costs include, but are not limited to, providing space within host cells for symbiont occupation (Douglas and Smith, 1983), creating and maintaining host-derived symbiosome membranes (Peng et al., 2010), actively concentrating carbon dioxide for symbiont photosynthesis (Weis et al., 1989; Meyer and Weis, 2012), detoxifying oxygen radicals, and repairing macromolecular damage caused by symbiont photo-oxidative stress (Lesser, 2006). The costs associated with each symbiont will cause the gross cost of symbiosis to increase with symbiont abundance (**Figure 2**). At high abundances, costs may increase exponentially, as carbon-limitation of symbiont photosynthesis may exacerbate photodamage and oxidative stress (Wooldridge, 2009; **Figure 2**). Importantly, the impact of these costs also depends on the amount of coral tissue incurring the cost, suggesting it may also be better predicted by adopting a symbiont to host ratio approach.

Thus, symbiont abundance may determine both the costs and benefits of symbiosis, which in turn determine the net benefit (or interaction outcome; **Figure 2**). The magnitude of this benefit may subsequently correlate with aspects of host performance, such that greater benefit facilitates faster growth or higher reproductive rates. This framework allows us to understand how variation in symbiont abundance may underlie variability in coral outcomes. For example, elevated nutrients have been shown to reduce coral growth (Marubini and Davies, 1996; Fabricius, 2005), which may reflect a nutrient-driven increase in symbiont abundance beyond the optimum that reduces the net benefit of symbiosis. We hypothesize that, if symbiosis costs and benefits are density-dependent, variation in symbiont abundance can help explain the natural variability observed in coral performance, both within and among coral species and colonies. This “density-dependent” model of coral-algal symbiosis provides a framework for generating and testing diverse hypotheses linking the environment to symbiont abundance, physiology, and function.

## EFFECT OF SYMBIONT ABUNDANCE ON SYMBIOSIS BREAKDOWN

Symbiont abundance can influence corals’ sensitivity to environmental stress and the breakdown of symbiosis that can occur as a result. Because photodamage and production of reactive oxygen species (ROS) in symbionts is thought to be the primary trigger of bleaching (Weis, 2008), this response should logically depend on symbiont abundance. However, a link between symbiont abundance and bleaching has only recently been shown: in the Pacific coral *Pocillopora damicornis,* colonies with more symbionts (measured by S/H cell ratios) bleached more severely in response to a natural warming event (Cunning and Baker, 2013), while higher S/H cell ratios were also linked to greater bleaching severity in experiments with the Caribbean corals *Montastraea cavernosa* (Silverstein et al., 2014), *Orbicella faveolata*, and *Siderastrea siderea* (Cunning, 2013) suggesting this may be a general phenomenon in corals. Although counter to previous suggestions that more symbionts (per cm^2^) may buffer corals from stress (Stimson et al., 2002; Enríquez et al., 2005), these findings are consistent with the molecular mechanisms of bleaching in suggesting that a larger symbiont pool produces more cumulative ROS, triggering a proportionally more severe bleaching response.

Under this model, if the primary sources and targets of ROS signaling are symbiont and host cells, respectively, then the S/H cell ratio may be the best predictor of the functional relationship between symbiont abundance and bleaching. In fact, areal symbiont abundance is suggested to have the opposite influence, such that fewer symbionts per unit area leads to reduced self-shading and greater light-driven ROS production per cell (Enríquez et al., 2005; Terán et al., 2010). However, cumulative ROS production, as the relevant metric in this framework, equals the per-cell rate times the total number of cells, and thus concomitant changes in both these factors must be evaluated to determine the net effect.

The relationship between symbiont abundance and local irradiance (i.e., self-shading, which may drive per-cell rates of ROS production) has been identified using both empirical and modeling approaches as being nonlinear, such that pigments (Enríquez et al., 2005) or symbionts (Terán et al., 2010) may decline by ∼80% before the internal light environment is significantly amplified. Consequently, large changes in symbiont abundance may take place without impacting light-driven ROS production per cell. Meanwhile, 80% fewer symbionts would reduce total ROS production by at least 80%, suggesting that corals with fewer symbionts may indeed experience less cumulative oxidative stress. However, enhanced ROS production per cell may become relatively more important if the symbiont pool is reduced below a threshold (e.g., due to partial bleaching) where the internal light environment becomes exponentially amplified (Enríquez et al., 2005; Terán et al., 2010). This positive feedback may accelerate coral bleaching in already-bleached corals, even though initial susceptibility may be greater when symbiont abundance is higher.

These hypotheses are supported by a study that used both area- and protein-normalized metrics to assess changes in symbiont abundance in two colonies of *Orbicella franksi* transplanted to a high light environment (Edmunds and Gates, 2002). Initial symbiont abundance per cm^2^ did not differ between colonies, but symbionts per mg protein differed by ∼60%. Only the coral with more symbionts per mg protein bleached when transplanted to the high light environment, supporting the hypothesis that excess symbionts cause more severe bleaching. Even though these corals showed different functional responses, areal symbiont density measurement failed to identify any difference between them, showing how certain metrics can mask or obscure important functional variation. This provides another illustration of how metrics that incorporate both symbiont and host information may be more relevant to physiology and better predict symbiosis functional outcomes.

## SYMBIONT ABUNDANCE VARIABILITY AND DYNAMICS

Understanding natural spatiotemporal variability in symbiont abundance is important due to the many ways it may influence symbiosis costs and benefits, coral performance, and stress susceptibility. Early studies found that symbiont abundance was partly determined by environmental conditions in *Hydra* (Douglas and Smith, 1984), *Aiptasia* (Steele, 1976), and corals (Dustan, 1979). In particular, these studies showed that differences in feeding and light regimes led to changes in symbiont abundance in the host. The apparent regulation of symbionts by the host was well-studied in *Hydra*, involving both arrested growth and expulsion of symbionts (Douglas and Smith, 1984). Corals may also actively regulate their symbiont populations, evidenced by continuous symbiont expulsion (Hoegh-Guldberg et al., 1987; Baghdasarian and Muscatine, 2000; Yamashita et al., 2011), and higher growth rates observed in *Symbiodinium* living outside the host (Chang et al., 1983). Various mechanisms of host control over the symbiont population have been investigated, including nutrient limitation (Falkowski et al., 1993), expulsion (Baghdasarian and Muscatine, 2000), apoptosis (Dunn and Weis, 2009), symbiophagy (Downs et al., 2009), and other mechanisms (Gates et al., 1992).

However, the underlying factors that determine the specific abundance of symbionts maintained by these mechanisms are not well understood (Douglas and Smith, 1984; Smith, 1987). It has been hypothesized that spatial or volumetric capacities determine the abundance of symbionts in a coral (Jones and Yellowlees, 1997), although changes in abundance on seasonal and diel scales and in response to abiotic factors (e.g., nutrients) suggest that mechanisms other than space-limitation are important (Davy et al., 2012). If corals actively regulate the size of their symbiont pool, it follows that they should maintain symbionts at an optimal abundance that maximizes the net benefit of the symbiosis (**Figure 2**; Hoogenboom et al., 2010; Cunning, 2013). This optimal abundance will be context-dependent, as abiotic factors such as light and temperature, and biotic factors such as symbiont type, are expected to influence the costs and benefits defining optimal abundance (**Figures 2A,B**). In this model, an optimal abundance exists for a given symbiont type in a given environment.

High spatial variability in abiotic factors, even over reefal scales (Brakel, 1979), may drive corresponding variation in optimal symbiont abundance, and regulation to match these variable optima may explain differences observed among coral colonies (Moothien-Pillay et al., 2005; Pisapia et al., 2014). Short- to mid-term temporal changes (days to weeks) in abiotic factors may similarly shift abundance optima, driving observed seasonal dynamics of symbiont populations (Stimson, 1997; Fagoonee et al., 1999; Fitt et al., 2000; Cunning and Baker, 2013). In this way, regulation by coral hosts to match dynamic optima that maximize interaction benefit may underlie observed spatiotemporal variability in symbiont abundance.

Alternative explanations for variation in symbiont abundance include direct environmental control of symbiont growth dynamics and the resulting differential performance of symbiont types with varying physiological optima. Additionally, the degree of symbiont regulation might also be expected to depend on the particular coral species and symbiont type involved, and might also be inhibited by certain abiotic factors (e.g., nutrients). In addition, some degree of time lag between changes in the environment and compensatory changes in symbiont population size might be expected. Consequently, even if hosts actively regulate symbiont populations, they may not always be maintained at optimal levels.

While the primary abiotic factors influencing symbiont abundance are likely to be light, temperature, and nutrients, other factors such as salinity, dissolved oxygen (Brown et al., 1999; Fagoonee et al., 1999), and pCO_2_ may also play important roles. These factors can be also incorporated into a density-dependent theoretical framework—by altering symbiosis costs and benefits and driving the need for host regulatory control. Additional data describing the effects of each of these factors on symbiont population dynamics, and their potential interactions, will help test this model.

In addition to the environmental factors that control symbiont abundance, biological factors may also be important drivers of symbiont standing stock. These factors include intrinsic differences in tissue architecture among coral species (i.e., corals with thinner tissues may have generally higher symbiont abundance relative to host tissue), reproductive status, and heterotrophy (see Implications Of Different Metrics Of Symbiont Abundance and **Figure 1**). In addition, lesions (due to parrotfish bites, physical impact, or partial mortality) can lead to reduced symbiont abundance in surrounding tissues (Palmer et al., 2011), and coral diseases can also destabilize symbiont abundance (Cervino et al., 2001; Toller et al., 2001). Differences in these biotic and abiotic factors within colonies and across reefs therefore may establish a wide range of symbiont abundance in coral tissues, even for corals of the same species hosting the same symbiont type. Different coral hosts with different algal symbionts only further increases natural variability in partner abundance on reefs.

## ECOLOGICAL IMPLICATIONS AND ENVIRONMENTAL CHANGE

We hypothesize that the complex and dynamic interaction between biotic and abiotic landscapes can give rise to significant spatiotemporal variability in symbiont abundance within corals and across reefs. Indeed, symbiont abundance in nearby colonies can vary from twofold to threefold (cells per cm^2^; Jones and Yellowlees, 1997; Moothien-Pillay et al., 2005) to 21-fold (S/H cell ratio; Cunning, 2013), and changes of similar magnitudes may occur seasonally within colonies (Thornhill et al., 2011; Cunning, 2013).

If partner abundance determines symbiotic interaction outcomes (net benefit), then variability in symbiont population size can translate to critical differences in coral holobiont performance. For example, in *P. damicornis*, variation in initial symbiont abundance drove high variability in bleaching response (0–77% reduction in S/H cell ratios in colonies hosting thermotolerant *Symbiodinium* D1, 46–95% in colonies hosting thermally sensitive C1b-c; Cunning and Baker, 2013). Variability in symbiont abundance may therefore help explain why bleaching is often patchy over relatively small scales, and even within single colonies (Rowan et al., 1997; Jones, 2008). Over larger scales, variability in symbiont abundance may explain why bleaching is more severe at certain locations (e.g., where abiotic conditions promote higher symbiont abundances), in certain coral species (McClanahan et al., 2004), or at different times of the year (e.g., summer, when S/H cell ratios are higher, Cunning and Baker, 2013). Thus, differences in symbiont abundance may help explain ecological patterns over many scales.

These relationships may also provide insight into the impacts of climate change, as the effects of a changing environment on reef coral ecology may be mediated by effects on symbiont abundance. For example, several studies have shown declines in areal symbiont densities in response to elevated pCO_2_, which has been interpreted as acidosis-induced coral bleaching (Anthony et al., 2008; Kaniewska et al., 2012). Alternatively, this response could be interpreted as a host-controlled reduction of symbiont abundance to sustain maximum interaction benefit in a high-pCO_2_ environment. Regardless, if corals under high pCO_2_ have fewer symbionts, they may be less susceptible to subsequent thermal stress due to lower cumulative ROS accumulation. This suggests that corals in naturally acidic areas, or at high latitudes where acidification may occur before warming (van Hooidonk et al., 2014), may be more bleaching resistant than conspecifics in different environments. If true, this would have important implications for survival trajectories of corals facing the combined effects of high temperature and pCO_2_. Testing this hypothesis will require acclimating corals to high pCO_2_ and allowing symbiont abundance to equilibrate to these conditions before applying thermal stress, in order to separate the effects of prior CO_2_ exposure from the effects of thermal stress.

Eutrophication is another factor affecting reefs worldwide that may interact with other stressors by influencing symbiont abundance. Excess nutrients can increase symbiont abundance by alleviating their normal state of nutrient limitation, which may cause the host to lose regulatory control of its symbionts (Falkowski et al., 1993), resulting in detrimental impacts on host growth and performance (Marubini and Davies, 1996; Fabricius, 2005). Moreover, enlarged symbiont populations may render nutrient-exposed corals more susceptible to thermal stress (Cunning and Baker, 2013; Vega Thurber et al., 2014). This indicates that efforts to reduce nutrient pollution on coral reefs may help corals be more resistant to climate change-related stressors (Wooldridge and Done, 2009; Cunning and Baker, 2013; Wiedenmann et al., 2013; Vega Thurber et al., 2014).

Bleaching susceptibility is not the only factor that may be affected by symbiont abundance. Because the magnitude of net benefit received by corals is also dependent on symbiont abundance (**Figures 2A,B**), important ecological parameters such as growth and reproduction may also be impacted. While these links must be quantified empirically, the mechanistic framework outlined here helps conceptualize and evaluate the links between environmental variability, symbiont population dynamics, and reef coral ecology.

## CONCLUSION

While much of the focus of recent research has been on the influence of symbiont identity, symbiont abundance must also be considered as a critical factor influencing the function of coral-algal symbioses. Efforts to evaluate coral responses to environmental stresses may therefore benefit from more rapid and accurate ways of measuring and monitoring symbiont abundance, not merely as a stress response, but as a critical metric of coral physiology that will help explain holobiont outcomes. Ideally, knowledge of both symbiont identity and abundance (with respect to both physical and biological units) would provide the most comprehensive information on the state of the symbiosis, but we suggest that taxon-specific symbiont to host cell ratios are currently the most biologically relevant and efficiently obtainable metrics. When applied to targeted symbiotic systems of interest they have shown consistent functional relationships with aspects of host performance such as bleaching severity, and may also be useful predictors of the overall costs and benefits of symbiosis. We suggest that the use of more relevant metrics and a greater appreciation for importance of symbiont abundance will advance our understanding of the biology of coral-algal symbioses and their responses to environmental change.

## Conflict of Interest Statement

The authors declare that the research was conducted in the absence of any commercial or financial relationships that could be construed as a potential conflict of interest.
